# Hormones Act in Concert to Direct Plant Growth

**DOI:** 10.1371/journal.pbio.0020299

**Published:** 2004-08-24

Anyone who thinks plants are passive inhabitants of their environment has never seen time-lapse footage of a seedling bursting from its protective shell or a climbing vine coiling around a tree. Such films dramatize a fundamental fact of plant life: survival depends on responding to environmental cues. Shoots grow toward light and against gravity. Stems and roots curl around obstacles that block their paths.

In plants, environmental cues trigger hormonal changes that in turn regulate cells' shapes and proliferation. In this way, subtle changes in the environment affect plant growth. Auxin, the first known plant hormone, spurs growth and shapes growth patterns in nearly every plant tissue throughout a plant's lifecycle. Brassinosteroids—a class of hormones chemically similar to animal steroids like testosterone—are linked to many of the same processes as auxin.[Fig pbio-0020299-g001]

**Figure pbio-0020299-g001:**
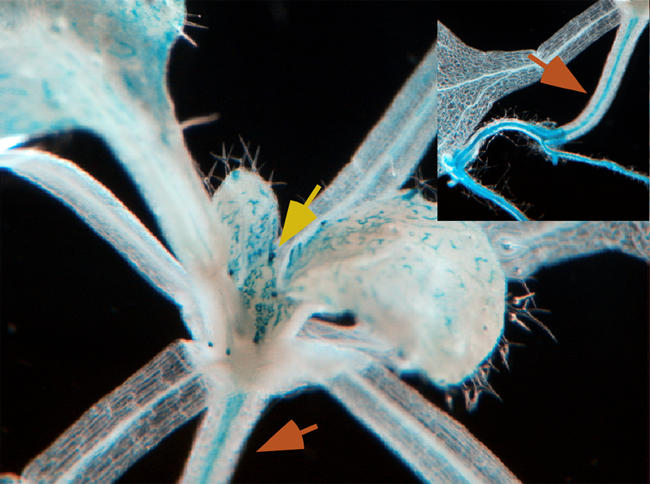
Emerging leaf tips (yellow arrow) and hypocotyl (orange arrows) of an Arabidopsis mutant

Early physiological and molecular experiments gave conflicting evidence about whether auxin and brassinosteroids had similar effects. For many years, biologists believed that these hormones acted through independent signal transduction pathways—chains of molecules that relay stimuli and elicit cellular responses, such as gene expression. But in the last few years, microarray studies, which can measure the transcription of thousands of genes simultaneously, showed that auxin and brassinosteroids do regulate expression of several genes in common.

In this issue of *PLoS Biology*, Jennifer Nemhauser et al. assay the entire genome of Arabidopsis thaliana, a favorite for plant genetics studies, for effects of auxin and brassinosteroids. The group's microarray analyses show that these hormones affect transcription of about 80 genes in common—including many known players in the hormones' signal transduction pathways. To see how this regulation could occur, the research team looked at the genes turned on by both hormones to find common promoter sequences—regions of the genome that do not code for protein but instead help regulate gene transcription. They used a new computational approach to tease out promoter regions that auxin and brassinosteroid pathways both act upon, showing how these hormones have overlapping effects on gene transcription.

The group also compared the effects of auxin and brassinosteroids on seedlings' stem growth and gene expression in a variety of mutant Arabidopsis lines. They showed that auxin and brassinosteroids greatly enhance each other's effects on stem growth, demonstrating that the interaction of these hormones is important for normal plant development. Mutants with a disabled auxin pathway don't respond normally to brassinosteroids, and vice versa. Also, mutants with abnormally high levels of auxin have a reduced number of genes that respond to brassinosteroids. Thus, these hormones act through overlapping, interdependent pathways—but they don't regulate each other directly. Instead, the researchers suggest, the pathways likely converge on the promoters of a few key genes.

It's still an open question why plants use these hormones with such redundant effects. Nemhauser speculates that—as is known to be the case in animals—by having dual, interdependent pathways, plants can finely tune how these ubiquitous hormones act in different cells and tissues to shape patterns of growth. By showing clearly that auxin and brassinosteroids act together and how they affect many of the same genes, Nemhauser and colleagues have set the stage for more detailed studies of how these hormones act in specific parts of plants to shape growth.

